# Validity of a Self-administered Food Frequency Questionnaire for Genomic and Omics Research Among Pregnant Women: The Tohoku Medical Megabank Project Birth and Three-Generation Cohort Study

**DOI:** 10.2188/jea.JE20240293

**Published:** 2025-07-05

**Authors:** Keiko Murakami, Misako Nakadate, Taku Obara, Misato Aizawa, Ippei Takahashi, Mami Ishikuro, Aoi Noda, Hisashi Ohseto, Noriyuki Iwama, Masatoshi Saito, Ribeka Takachi, Shiori Sugawara, Yudai Yonezawa, Takahiro Yamashita, Shigenori Suzuki, Junko Ishihara, Masayuki Yamamoto, Shinichi Kuriyama

**Affiliations:** 1 Tohoku Medical Megabank Organization, Tohoku University, Miyagi, Japan; 2 School of Life and Environmental Science, Azabu University, Kanagawa, Japan; 3 Graduate School of Medicine, Tohoku University, Miyagi, Japan; 4 Tohoku University Hospital, Miyagi, Japan; 5 Nara Women’s University Graduate School of Humanities and Sciences, Nara, Japan; 6 Department of Health and Nutrition, Sendai Shirayuri Women’s College, Miyagi, Japan; 7 Innovation Division, KAGOME Co., Ltd., Tochigi, Japan; 8 Graduate School of Environmental Health, Azabu University, Kanagawa, Japan; 9 International Research Institute of Disaster Science, Tohoku University, Miyagi, Japan

**Keywords:** food frequency questionnaire, genomic and omics research, Japan, pregnant women, validity

## Abstract

**Background:**

The Tohoku Medical Megabank Project has initiated the Birth and Three-Generation Cohort Study (TMM BirThree Cohort Study) including genomic and omics investigations and conducted a self-administered food frequency questionnaire with the response option “constitutionally unable to eat or drink it” for individual food items (TMM-FFQ) for pregnant women. This study evaluated the validity of the TMM-FFQ among pregnant women.

**Methods:**

Participants comprised 122 pregnant women aged ≥20 years residing in Miyagi Prefecture who completed weighed food records (WFRs) for 3 days as reference intake and the TMM-FFQ during mid-pregnancy. Correlations between nutrient or food group intakes based on the WFR and the TMM-FFQ were calculated using Spearman’s rank correlation coefficients (CCs), adjusting for energy intake and correcting for random within-individual variation of WFR. Cross-classification was also conducted according to quintiles using the WFR and TMM-FFQ data.

**Results:**

The percentages of participants who chose the “constitutionally unable to eat or drink it” option were >3% for seven food and drink items. CCs were >0.30 for 31 nutrients; the median across energy and 44 nutrients was 0.41. CCs were >0.30 for 14 food groups; the median across 20 food groups was 0.35. The median percentages of cross-classification into exact plus adjacent quintiles and extreme quintiles were 63.1% and 3.3% for energy and nutrients and 61.9% and 4.1% for food groups, respectively.

**Conclusion:**

The validity of the TMM-FFQ compared with the WFR was reasonable for certain nutrients and food groups among pregnant women in the TMM BirThree Cohort Study.

## INTRODUCTION

Pregnancy is a critical period in the life of the mother and her offspring, during which optimal maternal nutrition plays a key role in enhancing birth outcomes and the health of both the mother and child.^1^^–^^5^ Obtaining accurate measures of individual dietary intake is important for analyzing and evaluating the results of epidemiological studies on the association between diet and health. Food frequency questionnaires (FFQs) have been widely used for dietary assessment in epidemiological studies, because they assess habitual dietary intake over an extended period and are easy for participants to complete, often as a self-administered form.^6^ Each FFQ should be evaluated separately, because it may perform differently in different populations owing to cultural specificity and subtle changes in the design may affect its performance, even if it is largely based on a previous one.^7^

The Tohoku Medical Megabank Project (TMM), conducted by Tohoku University Tohoku Medical Megabank Organization (ToMMo) and Iwate Medical University Iwate Tohoku Medical Megabank Organization, has been developing a fundamental infrastructure consisting of genome and cohort information for precision medicine and personalized healthcare.^8^^–^^11^ The TMM has initiated the Birth and Three-Generation Cohort Study (TMM BirThree Cohort Study) with genomic and omics investigations throughout Miyagi Prefecture, including pregnant women and their families.^12^ This cohort study has included a self-administered FFQ (TMM-FFQ) for pregnant women, and has provided accumulating evidence that maternal diet during pregnancy is associated with birth outcomes and the health of both the mother and child.^13^^–^^18^ One unique aspect of the TMM-FFQ is the inclusion of the response option “constitutionally unable to eat or drink it” for individual food and drink items. This response option assesses the inability to consume a particular food or drink because of a physiological reaction to it and should be included in developing personalized nutrition strategies using genetic data, because it is important for the contribution of genetics to dietary behavior and nutrient metabolism.^19^^–^^21^ We previously demonstrated the validity of the TMM-FFQ among community-dwelling adults^22^; however, to the best of our knowledge, the validity of FFQs including this response option have never been evaluated among pregnant women.

Therefore, we aimed to evaluate the validity of the TMM-FFQ among pregnant women. The findings will contribute to the development of personalized nutrition strategies for pregnant women.

## METHODS

### Study population

Medical staff at 10 obstetric clinics and hospitals in Miyagi Prefecture, Japan, distributed leaflets about this validation study to pregnant women <25 weeks of gestation from February to December 2022. The leaflets were also posted on the ToMMo website and left at seven community support centers established by the ToMMo as local facilities for health assessment of cohort participants.^8^ The eligibility criteria of the present study were pregnant women aged ≥20 years who were residing in Miyagi Prefecture and who could complete the survey during mid-pregnancy (14–27 weeks of gestation). The ToMMo study office received calls from pregnant women who were interested in the study, explained the overview of the study, and sent the necessary documents and materials to the pregnant women after they confirmed the intention to participate. Pregnant women provided written informed consent to participate after watching explanatory videos and reading explanatory paper documents. The staff did not provide a face-to-face explanation because of the coronavirus disease 2019 (COVID-19) pandemic. A total of 126 pregnant women voluntarily agreed to participate in the study. Four women failed to complete the study because of their physical conditions or other difficulties, so 122 pregnant women who completed the study were included in the analyses. The protocol of the present study was reviewed and approved by the ToMMo Research Ethics Committee (2021-4-128) and all other collaborating research institutions.

### Dietary assessment

Participants were requested to answer the TMM-FFQ before the start of the food recording period. The TMM-FFQ consisted of 139 food and drink items in 10 frequency categories and three portion size categories. It asked respondents about their usual consumption of the listed foods and drinks from pregnancy awareness. The food list was initially developed and used for the Japan Public Health Center-based Prospective Study^23^^,^^24^ and subsequently modified.^22^^,^^25^ The frequency categories for food items were as follows: constitutionally unable to eat it, less than once/month or never, one to three times/month, once or twice/week, three or four times/week, five or six times/week, once/day, two or three times/day, four to six times/day, and at least seven times/day. Portion size was specified for each food item using three standard sizes: small (50% smaller than the standard amount), medium (standard amount), and large (50% larger than the standard amount). The frequency categories for drink items were as follows: constitutionally unable to drink it, less than once/week or never, once or twice/week, three or four times/week, five or six times/week, one cup/day, two or three cups/day, four to six cups/day, seven to nine cups/day, and at least 10 cups/day. If the staff identified missing answers or logical errors in the FFQ responses, they asked participants to provide that information again.

Participants were requested to complete weighed food records (WFRs) for 3 days, including two weekdays and one weekend day during mid-pregnancy (14–27 weeks of gestation). They received guidance on how to complete the WFRs by trained dietitians via explanatory videos, and measured food portions during meal preparation using supplied portable digital scales (Tanita Co., Ltd., Tokyo, Japan), spoons and cups. Participants recorded the approximate quantity of all foods in the meal and/or the names of the product and company for foods purchased or consumed outside the home. Trained dietitians checked the WFRs with the participants approximately the day after the 3-day WFRs via video call, in light of the COVID-19 pandemic. They coded foods and weights based on the Standard Tables of Food Composition in Japan 2010.^26^

The individual food items were classified into 20 predefined food groups, mainly according to the Standard Tables of Food Composition in Japan 2010.^22^^,^^26^ Intakes of energy, 44 nutrients, and 20 food groups were calculated using the Standard Tables of Food Composition in Japan 2010,^26^ the Standard Tables of Food Composition in Japan Fifth Revised and Enlarged Edition: Fatty Acids Section,^27^ and a specially developed food composition table for isoflavones and carotenoids in Japanese foods.^28^^–^^31^ Because a database for dietary supplements was not available, intake from dietary supplements was not included in calculations for either the FFQ or WFR.

### Other variables

Information on height, pre-pregnancy weight, morning sickness, smoking status, alcohol drinking, medication, and supplement use were collected using a self-administered questionnaire integrated with the FFQ. A history of morning sickness was measured using the question “Have you had any symptoms of morning sickness between this pregnancy and now?” with four response options: never, nausea only, vomited but could eat, or vomited and could not eat.^32^

### Statistical analysis

The mean intake of each nutrient and food group based on the FFQ was compared with that based on the WFR. Percentage differences were calculated for each nutrient and food group by dividing the difference in mean intake on the FFQ from that on the WFR by that on the WFR. The validity of the FFQ was determined using calculating Spearman’s rank correlation coefficients (CCs) between nutrient and food group intakes from the WFR and those from the FFQ for energy-adjusted values. A residual model was used for energy adjustment.^6^ CCs were corrected for the attenuating effect of random within-individual error.^6^ One systematic review of FFQs developed for pregnant women and one review of FFQs developed for the Japanese population used the following cutoff points for CCs: CCs ≥0.60 were classified as strong, 0.40–0.59 moderate, 0.30–0.39 fair, and <0.30 poor.^33^^,^^34^ The validity of the categorization was measured by calculating the number of participants classified into the same, adjacent, and extreme categories by cross-classification according to both quintiles using the WFR and the FFQ.^25^ Sensitivity analyses were conducted excluding participants who reported that they had vomited and could not eat because of morning sickness.

All analyses were conducted using SAS version 9.4 software (SAS Institute Inc., Cary, NC, USA).

## RESULTS

Table 1 shows the characteristics of the participants. Approximately half of the participants were 30–34 years old, and 9.0% had a pre-pregnancy body mass index ≥25.0 kg/m^2^. Approximately 90% of the participants had a history of morning sickness and 15.6% were suffering from morning sickness at the time of the study. None of the participants smoked at the time of the study and 4.9% quit after pregnancy awareness. Current drinkers accounted for 9.8%, and 8.2% were constitutionally unable to drink.

**Table 1.** Characteristics of participants (*n* = 122)

	*n*	(%)
Age		
<30 years	23	(18.9)
30–34 years	56	(45.9)
35–39 years	37	(30.3)
≥40 years	6	(4.9)
Pre-pregnancy body mass index		
<18.5 kg/m^2^	18	(14.8)
18.5–24.9 kg/m^2^	93	(76.2)
≥25.0 kg/m^2^	11	(9.0)
History of morning sickness		
Never	15	(12.3)
Nausea only	63	(51.6)
Vomited but could eat	36	(29.5)
Vomited and could not eat	8	(6.6)
Current symptoms of morning sickness		
Yes	19	(15.6)
No	103	(84.4)
Smoking status		
Current smoker	0	(0.0)
Quit after pregnancy awareness	6	(4.9)
Quit before pregnancy awareness	12	(9.8)
Never smoker	104	(85.3)
Alcohol drinking		
Current drinker	12	(9.8)
Past drinker	54	(44.3)
Never drinker	46	(37.7)
Constitutionally never drinker	10	(8.2)

Table 2 shows the percentages of participants who chose the response option “constitutionally unable to eat or drink it” for each food and drink item. The median percentage was 0.0%. The percentages were >3% for soy milk, papaya, eel, pork liver, chicken liver, low-fat milk, and canned coffee.

**Table 2.** The proportions of participants who chose the response option “constitutionally unable to eat or drink it” for each food and drink item (*n* = 122)

	*n*	%
Cereals		
Breads	0	(0.0)
Udon (thick wheat noodles)	0	(0.0)
Soba (buckwheat noodles)	1	(0.8)
Ramen (Chinese noodles)	0	(0.0)
Rice cake	0	(0.0)
Potatoes and starches		
Sweet potato	0	(0.0)
Potato	0	(0.0)
Taro	1	(0.8)
Konjac	0	(0.0)
Pulses		
Tofu for miso soup	0	(0.0)
Tofu for other dishes	0	(0.0)
Kori-dofu (freeze dried tofu)	0	(0.0)
Nama-age (fried slices of drained tofu)	0	(0.0)
Abura-age (fried thin slices of pressed tofu)	0	(0.0)
Natto (Fermented soybeans)	2	(1.6)
Soy milk	6	(4.9)
Nuts and seeds		
Peanuts	3	(2.5)
Vegetables		
Carrot	0	(0.0)
Spinach	0	(0.0)
Pumpkin	0	(0.0)
Cabbage	0	(0.0)
Japanese radish	0	(0.0)
Takuan-zuke (pickled with rice bran and salt)	0	(0.0)
Pickled green leafy vegetables	0	(0.0)
Pickled Chinese cabbage	0	(0.0)
Pickled cucumber	0	(0.0)
Pickled eggplant	0	(0.0)
Sweet pepper	0	(0.0)
Tomato	3	(2.5)
Welsh onion	0	(0.0)
Chinese chive	0	(0.0)
Garland chrysanthemum	2	(1.6)
Komatsuna (spinach mustard)	0	(0.0)
Broccoli	2	(1.6)
Onion	0	(0.0)
Cucumber	0	(0.0)
Eggplant	0	(0.0)
Chinese cabbage	0	(0.0)
Edible burdock	0	(0.0)
Bean sprout	0	(0.0)
Sayaingen (young kidney beans pods)	1	(0.8)
Lettuce	0	(0.0)
Tomato juice	3	(2.5)
Fruits		
Papaya	8	(6.6)
Mandarin	0	(0.0)
Other citrus fruits	0	(0.0)
Apple	0	(0.0)
Japanese persimmon	2	(1.6)
Strawberry	0	(0.0)
Grapes	0	(0.0)
Melon	3	(2.5)
Watermelon	2	(1.6)
Peach	0	(0.0)
Pear	0	(0.0)
Kiwifruit	2	(1.6)
Pineapple	1	(0.8)
Banana	0	(0.0)
Umeboshi (pickled plum)	3	(2.5)
Marmalade	0	(0.0)
100% orange juice beverage	0	(0.0)
100% apple juice beverage	0	(0.0)
Fungi		
Shiitake	1	(0.8)
Enokitake	0	(0.0)
Shimeji	1	(0.8)
Algae		
Wakame, kombu	0	(0.0)
Hijiki	0	(0.0)
Nori	0	(0.0)
Fish and shellfish		
Salted fish (Pacific cod, atka mackerel, salmon)	1	(0.8)
Dried fish	1	(0.8)
Canned tuna	0	(0.0)
Salmon, trout	1	(0.8)
Bonito, tuna	2	(1.6)
Japanese amberjack	1	(0.8)
Cod, flatfish	1	(0.8)
Sea bream	1	(0.8)
Horse mackerel, sardine	2	(1.6)
Pacific saury, mackerel	1	(0.8)
Shirasuboshi (boiled and dried whitebait)	1	(0.8)
Cod roe, salmon roe	2	(1.6)
Eel	4	(3.3)
Squid	1	(0.8)
Octopus	1	(0.8)
Shrimp	3	(2.5)
Short-neck clam, freshwater clam	1	(0.8)
Chikuwa (fish paste product)	1	(0.8)
Kamaboko (fish paste product)	1	(0.8)
Meats		
Beef, steak	1	(0.8)
Beef, grilled	0	(0.0)
Beef, stir-fried	0	(0.0)
Beef, stewed	0	(0.0)
Pork, stir-fried	1	(0.8)
Pork, deep-fried	1	(0.8)
Pork, stewed	1	(0.8)
Pork, simmered	2	(1.6)
Pork, in soup	1	(0.8)
Pork liver	10	(8.2)
Chicken, grilled	0	(0.0)
Chicken, stir-fried	0	(0.0)
Chicken, simmered	0	(0.0)
Chicken, deep-fried	0	(0.0)
Chicken liver	14	(11.5)
Loin ham	0	(0.0)
Sausage	0	(0.0)
Bacon	0	(0.0)
Eggs		
Egg	0	(0.0)
Milk and dairy products		
Low-fat milk	5	(4.1)
Milk	2	(1.6)
Cheese	0	(0.0)
Yogurt	0	(0.0)
Lactic acid bacteria beverage	0	(0.0)
Fats and oils		
Butter for spread	0	(0.0)
Margarine for spread	1	(0.8)
Confectionaries		
Japanese traditional confectionaries	1	(0.8)
Cake	0	(0.0)
Biscuit, cookie	0	(0.0)
Chocolate	0	(0.0)
Nonalcoholic beverages		
Green tea (sencha)	0	(0.0)
Green tea (bancha, genmaicha)	0	(0.0)
Oolong tea	0	(0.0)
Black tea	1	(0.8)
Coffee (not canned coffee)	2	(1.6)
Canned coffee	6	(4.9)
Soup	0	(0.0)
Soft drink	0	(0.0)
Energy drink	2	(1.6)
Seasonings and spices		
Dressing	0	(0.0)
Mayonnaise	1	(0.8)
Worcester sauce	0	(0.0)
Ketchup	0	(0.0)

**Median**	**0**	**(0.0)**

Table 3 shows energy and nutrient intakes from the WFR and FFQ, their percentage differences and correlations, and their comparison based on cross-classification by quintiles. The percentage difference in intakes between the WFR and FFQ varied from −37.6% for lycopene to 79.7% for β-cryptoxanthin. The deattenuated CCs ranged from 0.05 for sodium to 0.96 for daidzein and were >0.30 for 31 nutrients. The median across energy and 44 nutrients was 0.41. Classification into the same and adjacent categories for energy and nutrients ranged from 47.6% for monounsaturated fatty acid to 74.6% for calcium (median 63.1%). The proportion of participants classified into the opposite extreme category was ≤5% for most nutrients (median 3.3%). After excluding participants who had vomited and could not eat because of morning sickness (*n* = 8), the deattenuated CCs were >0.30 for 34 nutrients (median 0.40), and the median percentages of cross-classification into exact plus adjacent quintiles and extreme quintiles were 64.0% and 3.5%, respectively (eTable 1).

**Table 3.** Energy and nutrient intakes from the WFR and FFQ, their percentage differences and correlations, and their comparison based on cross-classification by quintile (*n* = 122)

	WFR		FFQ		%^a^	CC^b^	Cross-classification^c^		

							Same category	Same and adjacent category	Extreme category
	Mean (SD)		Mean (SD)				%	%	%
Energy, kcal	1,847	(346)	1,755	(555)	−5.0	0.16	23.0	60.7	4.1
Protein, g	68.8	(15.5)	61.4	(24.4)	−10.7	0.24	20.5	62.3	4.1
Total fat, g	65.2	(18.0)	62.7	(28.2)	−3.8	0.28	24.6	59.9	1.6
Carbohydrate, g	241.9	(45.4)	215.0	(58.7)	−11.1	0.43	24.6	61.5	1.6
Sodium, mg	3,444	(1,043)	3,174	(1,182)	−7.8	0.05	25.4	53.3	7.4
Potassium, mg	2,592	(705)	2,229	(897)	−14.0	0.44	30.3	68.9	2.5
Calcium, mg	564	(202)	567	(447)	0.5	0.55	33.6	74.6	2.5
Magnesium, mg	253	(70)	223	(86)	−11.7	0.61	39.4	73.8	1.6
Phosphorus, mg	1,049	(245)	1,006	(468)	−4.1	0.39	26.2	68.1	3.3
Iron, mg	7.9	(2.5)	6.7	(2.6)	−15.2	0.36	26.2	61.5	1.6
Zinc, mg	8.3	(1.9)	7.5	(2.9)	−9.8	0.21	23.0	60.7	3.3
Copper, mg	1.12	(0.26)	1.00	(0.35)	−10.6	0.44	27.9	64.8	4.1
Manganese, mg	2.79	(1.03)	2.24	(0.73)	−19.7	0.32	26.2	67.2	3.3
Retinol, µg	201	(269)	277	(245)	37.5	0.44	30.3	67.2	5.7
α-carotene, µg	514	(472)	506	(331)	−1.4	0.63	33.6	70.5	3.3
β-carotene, µg	3,125	(1,858)	2,547	(1,463)	−18.5	0.51	27.1	64.0	1.6
β-cryptoxanthin, µg	420	(865)	755	(884)	79.7	0.53	34.4	68.9	4.1
β-carotene equivalent, µg	3,977	(2,320)	3,189	(1,740)	−19.8	0.46	23.8	63.1	3.3
Retinol equivalent, µg	563	(352)	546	(272)	−3.0	0.34	25.4	60.7	3.3
Lycopene, µg	3,098	(3,926)	1,932	(3,088)	−37.6	0.46	32.0	68.9	4.9
Vitamin D, µg	5.3	(4.7)	5.4	(3.4)	1.8	0.31	23.8	64.8	6.6
α-tocopherol, mg	7.0	(2.1)	6.1	(2.7)	−12.5	0.33	27.9	60.7	4.9
β-tocopherol, mg	0.4	(0.1)	0.3	(0.1)	−20.9	0.34	23.0	63.1	5.7
γ-tocopherol, mg	10.4	(4.1)	9.6	(4.9)	−7.5	0.28	26.2	58.2	2.5
δ-tocopherol, mg	2.4	(1.1)	2.2	(1.2)	−7.6	0.44	27.1	60.7	4.1
Vitamin K, µg	242	(123)	233	(166)	−3.9	0.67	25.4	68.9	0.0
Vitamin B_1_, mg	1.02	(0.30)	0.87	(0.31)	−14.9	0.12	23.8	59.9	7.4
Vitamin B_2_, mg	1.30	(0.40)	1.22	(0.75)	−6.3	0.28	22.1	59.9	3.3
Niacin, mg	15.6	(5.2)	13.9	(5.7)	−10.7	0.29	26.2	60.7	3.3
Vitamin B_6_, mg	1.25	(0.37)	1.13	(0.42)	−9.6	0.37	31.2	68.1	4.9
Vitamin B_12_, µg	4.7	(3.5)	4.6	(2.8)	−2.0	0.51	24.6	69.7	4.9
Folate, µg	321	(112)	266	(115)	−17.1	0.48	32.0	60.7	1.6
Pantothenic acid, mg	6.36	(1.62)	6.37	(2.91)	0.2	0.47	32.8	73.0	4.1
Vitamin C, mg	111	(52)	86	(45)	−22.2	0.28	24.6	59.0	5.7
Daidzein, mg	10.8	(7.2)	14.0	(11.7)	29.6	0.96	35.3	68.9	0.0
Genistein, mg	18.0	(12.0)	23.3	(20.2)	29.7	0.90	32.0	68.1	0.8
Saturated fatty acids, g	20.18	(6.36)	21.09	(11.95)	4.5	0.49	33.6	67.2	4.1
Monounsaturated fatty acid, g	23.62	(7.50)	22.83	(9.97)	−3.3	0.08	19.7	47.6	4.1
Polyunsaturated fatty acid, g	12.13	(3.91)	11.16	(4.68)	−8.0	0.26	23.0	55.8	2.5
n-3 polyunsaturated fatty acids, g	1.85	(0.87)	1.71	(0.78)	−7.5	0.27	26.2	61.5	4.9
n-6 polyunsaturated fatty acids, g	10.18	(3.32)	9.41	(3.97)	−7.6	0.29	23.0	57.4	2.5
Cholesterol, mg	322	(126)	297	(250)	−7.7	0.41	24.6	63.1	4.1
Dietary fiber, g	14.7	(4.3)	10.6	(4.5)	−27.6	0.49	27.9	73.0	3.3
Soluble dietary fiber, g	3.7	(1.3)	2.6	(1.2)	−28.2	0.47	27.1	65.6	2.5
Insoluble dietary fiber, g	10.4	(3.0)	7.5	(3.1)	−27.3	0.50	34.4	67.2	3.3

**Median**						**0.41**	**26.2**	**63.1**	**3.3**

CC, correlation coefficient; FFQ, food frequency questionnaire; SD, standard deviation; WFR, weighed food record.

^a^Percentage differences: (FFQ − WFR)/WFR × 100 (%).

^b^Spearman’s rank CCs based on energy-adjusted values and expressed as deattenuated CC. Deattenuated CCx = observed CCx * SQRT (1 + λx/n), where λx is the ratio of within-individual to between-individual variance for energy and nutrient x, and n is the number of WFR.

^c^Percentages were calculated according to cross-classification by quintiles based on energy-adjusted intakes.

Table 4 shows intakes for food groups from the WFR and FFQ, their percentage differences and correlations, and their comparison based on cross-classification by quintiles. The percentage difference in intakes between the WFRs and the FFQ ranged from −90.9% for sugar to 39.4% for milk and dairy products. The deattenuated CCs ranged from 0.04 for sugar to 0.75 for milk and dairy products and were >0.30 for 14 food groups. The median across 20 food groups was 0.35. Classification into the same and adjacent categories for food groups ranged from 50.0% for sugar to 77.1% for milk and dairy products (median 61.9%). The proportion of participants classified into the opposite extreme category was ≤5% for most food groups (median 4.1%). After excluding participants who had vomited and could not eat because of morning sickness (*n* = 8), the deattenuated CCs were >0.30 for 15 food groups (median 0.40), and the median percentages of cross-classification into exact plus adjacent quintiles and extreme quintiles were 59.7% and 4.4%, respectively (eTable 2).

**Table 4.** Food group intakes from the WFR and FFQ, their percentage differences and correlations, and their comparison based on cross-classification by quintile (*n* = 122)

	WFR		FFQ		%^a^	CC^b^	Cross-classification^c^		

							Same category	Same and adjacent category	Extreme category
	Mean (SD)		Mean (SD)				%	%	%
Cereals, g	350.9	(100.2)	416.8	(116.7)	18.8	0.44	29.5	59.9	2.5
Potatoes and starches, g	44.8	(42.4)	25.2	(22.1)	−43.8	0.32	25.4	59.0	7.4
Sugar, g	8.2	(6.4)	0.7	(5.5)	−90.9	0.04	19.7	50.0	8.2
Pulses, g	60.6	(61.1)	63.3	(70.0)	4.4	0.69	31.2	68.9	2.5
Nuts and seeds, g	3.0	(4.4)	0.9	(2.2)	−68.4	0.15	20.5	59.0	7.4
Vegetables, g	270.0	(122.8)	157.4	(106.4)	−41.7	0.43	28.7	59.9	4.1
Green and yellow vegetables, g	120.9	(79.8)	68.3	(54.7)	−43.5	0.43	33.6	65.6	4.1
White vegetables, g	149.1	(84.7)	89.1	(65.3)	−40.3	0.20	25.4	56.6	4.9
Pickled vegetables, g	5.1	(9.1)	5.1	(7.6)	−0.2	0.33	21.3	55.8	3.3
Fruits, g	129.1	(96.1)	166.5	(138.2)	28.9	0.46	31.2	64.0	4.9
Fungi, g	16.6	(15.8)	11.4	(10.5)	−31.6	0.44	30.3	62.3	3.3
Algae, g	6.9	(15.4)	6.1	(6.8)	−12.0	0.37	25.4	62.3	3.3
Fish and shellfish, g	38.0	(33.1)	35.3	(24.8)	−7.1	0.30	20.5	61.5	6.6
Meats, g	98.8	(44.7)	80.8	(41.7)	−18.2	0.24	25.4	63.1	6.6
Eggs, g	36.0	(24.6)	37.2	(52.0)	3.2	0.46	27.1	62.3	3.3
Milk and dairy products, g	184.9	(127.4)	257.7	(351.4)	39.4	0.75	35.3	77.1	1.6
Fats and oils, g	12.7	(6.7)	10.1	(4.9)	−20.4	0.14	23.8	54.1	4.9
Confectionaries, g	41.2	(35.2)	23.0	(29.6)	−44.1	0.56	30.3	71.3	4.1
Nonalcoholic beverages, g	566.4	(410.6)	213.0	(208.8)	−62.4	0.20	21.3	53.3	3.3
Seasonings and spices, g	92.9	(59.2)	18.4	(9.9)	−80.2	0.33	27.1	68.9	5.7

**Median**						**0.35**	**26.2**	**61.9**	**4.1**

CC, correlation coefficient; FFQ, food frequency questionnaire; SD, standard deviation; WFR, weighed food record.

^a^Percentage differences: (FFQ − WFR)/WFR × 100 (%).

^b^Spearman’s rank CCs based on energy-adjusted values and expressed as deattenuated CC. Deattenuated CCx = observed CCx * SQRT (1 + λx/n), where λx is the ratio of within-individual to between-individual variance for food group x, and n is the number of WFR.

^c^Percentages were calculated according to cross-classification by quintiles based on energy-adjusted intakes.

## DISCUSSION

The present study examined the validity of nutrient and food group intakes from the TMM-FFQ developed for genomic and omics research and containing the response option “constitutionally unable to eat or drink it” among pregnant women. Although the median percentage of participants who chose this response option was 0.0%, the percentages were not negligible for some food and drink items. The validity of the TMM-FFQ compared with the WFR was reasonable for certain nutrients and food groups. The agreement rates based on cross-classification by quintiles for nutrients and food groups were acceptable.

The median CCs between intakes from the WFR and TMM-FFQ were 0.41 for energy and 44 nutrients and 0.35 for 20 food groups. One systematic review of the FFQs developed for pregnant women demonstrated that about half of FFQs showed moderate or high validity for nutrients, with mean CCs of ≥0.40.^33^ One validation study of the FFQ among Japanese pregnant women found that CCs for a number of nutrients and food groups were lower than those reported in other studies of the FFQs among pregnant women.^35^ These results suggest that the validity of FFQs among Japanese pregnant women was not high compared with that reported in other countries. This may be because it is difficult to assess complicated modern Japanese diets accurately, because Japanese tend to consume a variety of foods such as grain, vegetables, fruits, fish and meat, and milk dishes.^36^ However, the CCs were >0.30 for 31 nutrients and 14 food groups. In addition, the median percentages of cross-classification into exact plus adjacent quintiles were >60.0% and those of cross-classification into extreme categories were only <5.0% for nutrients and food groups.

One review of FFQs developed for Japanese populations showed that the median CCs between dietary records and FFQs ranged from 0.31–0.56 for nutrients.^34^ One validation study of the FFQ showed that the median CCs between 4-day WDRs and FFQ were 0.47 for 45 nutrients and 0.51 for 43 food groups among middle-aged female cancer screenees living in urban areas in Japan.^25^ The TMM-FFQ was developed based on the above FFQ. We previously demonstrated that the median CC for food groups between the 12-day WDRs and TMM-FFQ was 0.45 among non-pregnant women aged ≥20 years living in Miyagi Prefecture.^22^ These results suggest that the validity of FFQs among pregnant women was comparatively lower than that reported among non-pregnant women in Japan. There are two possible reasons for this lower validity. First, assessing dietary intake among pregnant women is complicated because of various factors that are dependent on the period of gestation.^37^ A poor correlation between instruments may be partly explained by appetite fluctuations and nausea.^37^ This possible reason is supported by the present findings, in which the median CC increased from 0.35 to 0.40 across food groups after excluding participants who had vomited and could not eat because of morning sickness, although the median CC slightly decreased from 0.41 to 0.40 across energy and nutrients. The TMM-FFQ may not be suitable for food group intakes among pregnant women who vomit and cannot eat because of morning sickness. Second, the WFR only covered 3 days in the present study, although it is a common method for the validation study of FFQs among pregnant women,^33^ and CCs were corrected for within-individual variation in the WFRs. The CCs increased as the number of collection days increased in reference methods, indicating a higher correlation with a greater amount of data collected.^7^ Especially for food groups, the lower CCs may have been caused by the fact that some participants did not consume them in the 3-day WFR period, because day-to-day variation in the intake of most specific foods is substantially greater than day-to-day variation in nutrient intakes.^6^ Efforts to increase the duration of recording in the reference method would provide a better measure of habitual intake, which is generally more similar to the type of information generated by FFQs.^7^

The median percentage of participants who chose the response option “constitutionally unable to eat or drink it” was 0.0%, but the percentages were >3% for soy milk (4.9%), papaya (6.6%), eel (3.3%), pork liver (8.2%), chicken liver (11.5%), low-fat milk (4.1%), and canned coffee (4.9%). We previously found that the median percentage among non-pregnant women aged ≥20 years living in Miyagi Prefecture was 0.8%, and the percentages were >3% for 21 food items and high especially for papaya (8.1%), eel (7.3%), pork liver (8.9%), chicken liver (12.1%), low-fat milk (6.5%), margarine for spread (6.5%), alcoholic beverages (12.1%), and canned coffee (8.9%).^22^ These results suggest that pregnant women were less likely to choose this response option, but chose this response for similar food items to non-pregnant women. We also previously showed the validity of food groups including foods with high percentages of this option.^22^ Considering these situations, this response option did not seem to affect the validity of the FFQ.

CCs ranged from 0.05 for sodium to 0.96 for daidzein and from 0.04 for sugar to 0.75 for milk and dairy products. The variations may partly reflect differences in the number of food items, the ability to recall intake frequencies and portion sizes, the wording of FFQ questions, and between-individual variation in the consumption of different food groups.^34^ Pregnant women require additional iron and folate to meet their own nutritional needs as well as those of the developing fetus, because iron and folate deficiencies during pregnancy may have negative impacts on the health of the mother, her pregnancy, as well as fetal development.^38^ Dietary guidelines for pre-pregnant, pregnant, and lactating women in Japan also recommend increased dietary intakes of iron and folate, because Japanese women tend to have insufficient intakes of these nutrients.^39^ The TMM-FFQ showed fair validity for iron and moderate validity for folate. The TMM-FFQ also showed moderate validity for confectionaries, with a CC of 0.56. The validation study of the TMM-FFQ among non-pregnant women showed that the CC for confectionaries was 0.35,^22^ and one review of FFQs developed in Japan gave a value of 0.38.^34^ One possible reason for this relatively high CC among pregnant women is that they tend to remember how much confectionaries they have consumed, because they are conscious about gestational weight gain.^40^ One review of pregnancy diet in the Avon Longitudinal Study of Parents and Children found the most robust association between maternal intakes of fish and shellfish and neurocognitive development in the offspring, with fish consumption being beneficial to childhood outcomes.^41^ The TMM-FFQ showed a CC of 0.30 for fish and shellfish; however, this value increased to 0.41 after excluding participants who had vomited and could not eat because of morning sickness. Differences in the validity of nutrients and food groups in FFQs among different populations should be considered when comparing health effects among nutrients and food groups.

The present study had several limitations. First, WFRs can be susceptible to measurement errors caused by erroneous recording, although we assumed that nutrient and food group intakes from the WFR were the gold standard. Nevertheless, WFRs have the least correlated errors with FFQs because portions are weighed.^7^ Second, the generalizability of the present findings should be cautioned because participants were not selected randomly. In addition, participants may have been highly health conscious, because they all completed the study despite its strict design. Finally, the reason why participants selected the response option “constitutionally unable to eat or drink it” is unclear. Some may have chosen this option because of physical reactions, whereas others may have chosen the option just because of taste preferences. However, participants were instructed to choose the “less than once/month or never” option for foods and the “less than once/week or never” option for drinks if they did not consume specific foods and drinks for reasons other than constitutional ones.

In conclusion, this study demonstrated that the TMM-FFQ with the response option “constitutionally unable to eat or drink it” can be considered suitable at least for assessing intakes of certain nutrients and food groups among pregnant women in the TMM BirThree Cohort Study.

## SUPPLEMENTARY MATERIAL

eTable 1. Energy and nutrient intakes from the WFR and FFQ, their percentage differences and correlations, and their comparison based on cross-classification by quintile, excluding participants who had vomited and could not eat because of morning sickness (n = 114)

eTable 2. Food group intakes from the WFR and FFQ, their percentage differences and correlations, and their comparison based on cross-classification by quintile, excluding participants who had vomited and could not eat because of morning sickness (n = 114)
