# Supplementary material for: Validity of a Self-administered Food Frequency Questionnaire for Genomic and Omics Research Among Pregnant Women: The Tohoku Medical Megabank Project Birth and Three-Generation Cohort Study
Source: J Epidemiol. 2025 Jul 5;35(7):305–12. doi: 10.2188/jea.JE20240293 (PMC12162182; doi:10.2188/jea.JE20240293)
Supplement: Supplementary file 1 [file je-35-305-s001.pdf]

eTable 1. Energy and nutrient intakes from the WFR and FFQ, their percentage differences and correlations, and their comparison based on cross-classification by quintile, excluding participants who had vomited and could not eat because of morning sickness (n=114)

	WFR		FFQ		%[a]	CC[b]	Cross-classification[c]		
							Same category %	Same and adjacent category %	Extreme category %
	Mean	(SD)	Mean	(SD)					
Energy, kcal	1,863	(330)	1,775	(564)	−4.8	0.11	20.2	61.4	4.4
Protein, g	69.3	(15.2)	62.3	(24.8)	−10.0	0.29	24.6	65.8	3.5
Total fat, g	65.8	(17.5)	63.6	(28.7)	−3.3	0.31	25.4	57.9	1.8
Carbohydrate, g	244.1	(44.4)	217.5	(58.7)	−10.9	0.47	23.7	63.2	1.8
Sodium, mg	3,476	(1,051)	3,203	(1,198)	−7.9	−0.02	21.1	50.0	7.9
Potassium, mg	2,616	(697)	2,262	(910)	−13.6	0.42	30.7	66.7	2.6
Calcium, mg	571	(198)	582	(458)	1.9	0.56	28.1	75.4	2.6
Magnesium, mg	257	(70)	227	(88)	−11.6	0.62	38.6	72.8	1.8
Phosphorus, mg	1,059	(239)	1,024	(477)	−3.3	0.38	28.1	66.7	3.5
Iron, mg	8.0	(2.5)	6.8	(2.7)	−15.3	0.37	25.4	60.5	1.8
Zinc, mg	8.4	(1.8)	7.6	(2.9)	−9.3	0.21	22.8	60.5	2.6
Copper, mg	1.14	(0.25)	1.02	(0.36)	−10.4	0.45	30.7	65.8	4.4
Manganese, mg	2.83	(1.02)	2.26	(0.75)	−20.3	0.33	28.1	68.4	3.5
Retinol, μg	206	(277)	276	(247)	34.4	0.49	29.8	70.2	6.1
α-carotene, μg	524	(483)	519	(334)	−0.9	0.60	34.2	67.5	3.5
β-carotene, μg	3,190	(1,885)	2,586	(1,480)	−18.9	0.51	28.1	66.7	3.5
β-cryptoxanthin, μg	407	(860)	762	(900)	87.2	0.51	32.5	68.4	4.4
β-carotene equivalent, μg	4,023	(2,362)	3,239	(1,755)	−19.5	0.46	24.6	63.2	3.5
Retinol equivalent, μg	569	(359)	549	(275)	−3.5	0.35	21.9	58.8	2.6
Lycopene, μg	3,110	(3,982)	1,942	(3,145)	−37.6	0.42	35.1	69.3	6.1
Vitamin D, μg	5.2	(4.7)	5.5	(3.5)	4.8	0.28	26.3	64.0	7.0
α-tocopherol, mg	7.1	(2.1)	6.2	(2.7)	−13.1	0.27	24.6	57.9	5.3
β-tocopherol, mg	0.4	(0.1)	0.3	(0.1)	−21.4	0.33	22.8	60.5	7.0
γ-tocopherol, mg	10.7	(3.9)	9.7	(5.0)	−9.3	0.34	23.7	58.8	2.6
δ-tocopherol, mg	2.5	(1.1)	2.2	(1.3)	−8.8	0.48	26.3	60.5	4.4
Vitamin K, μg	245	(125)	236	(171)	−3.7	0.66	28.9	68.4	0.0
Vitamin B_1, mg	1.03	(0.30)	0.88	(0.31)	−14.1	0.10	21.9	59.7	7.9
Vitamin B_2, mg	1.30	(0.39)	1.24	(0.77)	−4.3	0.30	24.6	60.5	4.4
Niacin, mg	15.6	(5.1)	14.1	(5.8)	−9.8	0.31	29.8	62.3	2.6

Vitamin B_6, mg	1.26	(0.37)	1.15	(0.42)	−9.4	0.35	30.7	66.7	5.3
Vitamin B_{12}, μg	4.6	(3.5)	4.6	(2.8)	−0.4	0.52	27.2	69.3	5.3
Folate, μg	326	(113)	269	(116)	−17.5	0.45	33.3	60.5	1.8
Pantothenic acid, mg	6.40	(1.60)	6.48	(2.96)	1.2	0.46	34.2	72.8	3.5
Vitamin C, mg	112	(52)	86	(44)	−23.1	0.23	24.6	57.0	6.1
Daidzein, mg	10.9	(7.3)	14.3	(12.0)	31.4	0.96	35.1	68.4	0.0
Genistein, mg	18.1	(12.2)	23.8	(20.7)	31.6	0.91	31.6	70.2	0.0
Saturated fatty acids, g	20.35	(6.21)	21.42	(12.19)	5.2	0.55	34.2	67.6	3.5
Monounsaturated fatty acid, g	23.83	(7.31)	23.11	(10.13)	−3.0	0.14	18.4	50.9	2.6
Polyunsaturated fatty acid, g	12.34	(3.76)	11.27	(4.76)	−8.7	0.28	22.8	60.5	2.6
n-3 polyunsaturated fatty acids, g	1.86	(0.86)	1.73	(0.80)	−7.4	0.25	26.3	57.9	5.3
n-6 polyunsaturated fatty acids, g	10.38	(3.22)	9.50	(4.03)	−8.4	0.32	21.9	60.5	2.6
Cholesterol, mg	323	(124)	303	(257)	−6.2	0.40	24.6	64.0	4.4
Dietary fiber, g	14.9	(4.3)	10.7	(4.5)	−27.8	0.46	24.6	72.8	3.5
Soluble dietary fiber, g	3.7	(1.3)	2.7	(1.2)	−28.0	0.42	24.6	63.2	2.6
Insoluble dietary fiber, g	10.5	(3.0)	7.6	(3.1)	−27.6	0.47	32.5	66.7	3.5
**Median**						**0.40**	**26.3**	**64.0**	**3.5**

CC, correlation coefficient; FFQ, food frequency questionnaire; SD, standard deviation; WFR, weighed food record.

[a]Percentage differences: (FFQ − WFR)/WFR×100 (%).

[b]Spearman's rank CCs based on energy-adjusted values and expressed as deattenuated CC. Deattenuated CCx = observed CCx * SQRT (1 + λx/n), where λx is the ratio of within-individual to between-individual variance for energy and nutrient x, and n is the number of WFR.

[c]Percentages were calculated according to cross-classification by quintiles based on energy-adjusted intakes.

eTable 2. Food group intakes from the WFR and FFQ, their percentage differences and correlations, and their comparison based on cross-classification by quintile, excluding participants who had vomited and could not eat because of morning sickness (n=114)

	WFR		FFQ		%[a]	CC[b]	Cross-classification[c]		
							Same category	Same and adjacent category	Extreme category
	Mean	(SD)	Mean	(SD)			%	%	%
Cereals, g	353.5	(100.7)	421.3	(117.1)	19.2	0.41	28.1	59.6	2.6
Potatoes and starches, g	44.0	(39.0)	25.3	(22.7)	−42.5	0.40	28.1	60.5	7.0
Sugar, g	8.5	(6.3)	0.7	(5.6)	−91.5	0.19	17.5	50.0	7.0
Pulses, g	62.6	(62.5)	64.5	(72.0)	3.0	0.73	30.7	68.4	1.8
Nuts and seeds, g	3.1	(4.5)	1.0	(2.3)	−68.6	0.11	21.9	58.8	7.0
Vegetables, g	276.0	(123.5)	158.3	(107.0)	−42.6	0.40	27.2	58.8	4.4
Green and yellow vegetables, g	121.8	(80.8)	68.3	(54.5)	−43.9	0.48	29.8	68.4	5.3
White vegetables, g	154.2	(84.7)	90.0	(66.2)	−41.6	0.18	24.6	56.1	6.1
Pickled vegetables, g	5.1	(9.3)	5.2	(7.7)	3.0	0.39	20.2	57.9	0.9
Fruits, g	129.7	(98.4)	166.2	(140.4)	28.2	0.44	25.4	64.9	5.3
Fungi, g	16.7	(15.8)	11.4	(10.6)	−31.5	0.41	28.1	62.3	3.5
Algae, g	6.8	(15.3)	6.1	(7.0)	−9.5	0.38	25.4	59.7	3.5
Fish and shellfish, g	37.8	(33.3)	35.4	(25.3)	−6.1	0.41	17.5	58.8	4.4
Meats, g	99.3	(43.8)	81.2	(41.6)	−18.3	0.32	24.6	64.0	5.3
Eggs, g	36.3	(24.9)	38.3	(53.5)	5.3	0.50	27.2	59.7	2.6
Milk and dairy products, g	187.2	(128.6)	267.3	(361.3)	42.8	0.86	40.4	80.7	1.8
Fats and oils, g	13.0	(6.7)	10.1	(5.0)	−22.2	0.18	26.3	57.0	4.4
Confectionaries, g	41.4	(33.6)	23.1	(30.0)	−44.3	0.58	29.8	70.2	3.5
Nonalcoholic beverages, g	573.4	(414.6)	210.6	(212.5)	−63.3	0.17	19.3	52.7	4.4
Seasonings and spices, g	92.6	(59.0)	18.8	(10.2)	−79.7	0.38	27.2	66.7	3.5
**Median**						**0.40**	**26.8**	**59.7**	**4.4**

CC, correlation coefficient; FFQ, food frequency questionnaire; SD, standard deviation; WFR, weighed food record.
[a]Percentage differences: (FFQ − WFR)/WFR×100 (%).
[b]Spearman's rank CCs based on energy-adjusted values and expressed as deattenuated CC. Deattenuated CCx = observed CCx * SQRT (1 + λx/n), where λx is the ratio of within-individual to between-individual variance for food group x, and n is the number of WFR.
[c]Percentages were calculated according to cross-classification by quintiles based on energy-adjusted intakes.
